# Supplementary material for: Modelling the impact of combining HIV prevention interventions on HIV dynamics in fishing communities in Uganda
Source: BMC Infect Dis. 2023 Mar 22;23:173. doi: 10.1186/s12879-023-08113-2 (PMC10031877; doi:10.1186/s12879-023-08113-2)
Supplement: Supplementary file 1 — Supplementary Material 1 [file 12879_2023_8113_MOESM1_ESM.pdf]

Additional File 1: Detailed description of the compartmental model - Supplementary Tables and Figures

Contents

A Model description **1**

B Supplementary Tables **12**

C Supplementary Figures **17**

A Model description

We developed a population-level, deterministic, compartmental model of heterosexual HIV transmission in the population aged 16-60 years, stratified by gender (r) and sexual risk group (j). Susceptible individuals are either not receiving PrEP (S^1) or receiving PrEP (S^2), with additional compartments for circumcised males (not receiving PrEP, S^3, or receiving PrEP, S^4). A proportion p_c of males is assumed to enter the susceptible population already circumcised. Once infected, individuals are assumed to move through four stages of disease progression: (i) an initial high viral (HV) load stage (H), (ii) a low viral (LV) load stage (Y), (iii) a pre-AIDS high VL stage (Z), and (iv) a final AIDS stage (A). When tested (according to a rate of diagnosis τ^T such that the proportion of infected individuals aware of their status increases linearly over time), infected individuals move to the corresponding 'diagnosed' compartment (representing awareness of HIV+ status) in which they are assumed to (slightly) reduce their risk behaviour due to HIV counseling. Infected individuals that have been tested and hence are aware of their disease status initiate ART with a certain probability p_{ART} (increasing over time) depending on their disease stage. ART dropout is also modelled and includes treatment failure

(i.e., individuals move back to the untreated compartment corresponding to their disease stage). Individuals that have dropped out of ART may then restart treatment with the same probability as previously untreated individuals. Susceptible individuals that have been tested but are not infected initiate PrEP with a certain probability p_{PrEP} that is increasing over time from 2015 onwards [1]. PrEP discontinuation ω and ART dropout γ are modeled as fixed annual rates. Figure S1 displays the model structure for males in risk group j. For females, the model structure is equivalent but without the leftmost 'circumcised' compartments. We assume HIV testing to be available since 1987 [2], and ART since 2004 for individuals in the (pre-)AIDS stage [3] and since 2011 for all stages [1]. Intervention uptake in males is assumed to be lower than in females [4].

The model parameters and their uncertainty ranges are shown in Table S2. Let subscripts r' and p denote the gender and risk group of a partner of an individual from subgroup (r, j). The model is described by the following set of ordinary differential equations (for females), where N_{rj} is the total number of individuals in subgroup (r, j) and π^x_{rj} is the force of infection.

$$\frac{dS^1_{rj}}{dt} = (\mu + \psi)N_{rj} + \omega S^2_{rj} - (\pi^1_{rj} + \theta + \psi)S^1_{rj}$$

$$\frac{dS^2_{rj}}{dt} = \theta S^1_{rj} - (\pi^2_{rj} + \omega + \psi)S^2_{rj}$$

$$\frac{dH^1_{rj}}{dt} = \pi^1_{rj}S^1_{rj} + \pi^2_{rj}S^2_{rj} - (\nu^1 + \tau^T + \psi)H^1_{rj}$$

$$\frac{dH^2_{rj}}{dt} = \tau^T H^1_{rj} + \gamma H^3_{rj} - (\nu^1 + \tau^A_1 + \psi)H^2_{rj}$$

$$\frac{dH^3_{rj}}{dt} = \tau^A_1 H^2_{rj} - (\nu^2 + \gamma + \psi)H^3_{rj}$$

$$\frac{dY^1_{rj}}{dt} = \nu^1 H^1_{rj} - (\Omega^1 + \tau^T + \psi)Y^1_{rj}$$

$$\frac{dY^2_{rj}}{dt} = \nu^1 H^2_{rj} + \tau^T Y^1_{rj} + \gamma Y^3_{rj} - (\Omega^1 + \tau^A_2 + \psi)Y^2_{rj}$$

$$\frac{dY^3_{rj}}{dt} = \nu^2 H^3_{rj} + \tau^A_2 Y^2_{rj} - (\Omega^2 + \gamma + \psi)Y^3_{rj}$$

$$\frac{dZ^1_{rj}}{dt} = \Omega^1 Y^1_{rj} - (\Psi^1 + \tau^T + \psi)Z^1_{rj}$$

$$\frac{dZ^2_{rj}}{dt} = \Omega^1 Y^2_{rj} + \tau^T Z^1_{rj} + \gamma Z^3_{rj} - (\Psi^1 + \tau^A_3 + \psi)Z^2_{rj}$$

$$\frac{dZ^3_{rj}}{dt} = \Omega^2 Y^3_{rj} + \tau^A_3 Z^2_{rj} - (\Psi^2 + \gamma + \psi)Z^3_{rj}$$

$$\frac{dA^1_{rj}}{dt} = \Psi^1 Z^1_{rj} - (\delta^1 + \tau^T + \psi)A^1_{rj}$$

$$\frac{dA^2_{rj}}{dt} = \Psi^1 Z^2_{rj} + \tau^T A^1_{rj} + \gamma A^3_{rj} - (\delta^1 + \tau^A_4 + \psi) A^2_{rj}$$

$$\frac{dA^3_{rj}}{dt} = \Psi^2 Z^3_{rj} + \tau^A_4 A^2_{rj} - (\delta^2 + \gamma + \psi) A^3_{rj}$$

For males, the equations below differ from those for females due to the inclusion of circumcision compartments.

$$\frac{dS^1_{rj}}{dt} = (1 - p_c)(\mu + \psi) N_{rj} + \omega S^2_{rj} - (\pi^1_{rj} + \sigma + \theta + \psi) S^1_{rj}$$

$$\frac{dS^2_{rj}}{dt} = \theta S^1_{rj} - (\pi^2_{rj} + \sigma + \omega + \psi) S^2_{rj}$$

$$\frac{dS^3_{rj}}{dt} = p_c(\mu + \psi) N_{rj} + \sigma S^1_{rj} + \omega S^4_{rj} - (\pi^3_{rj} + \theta + \psi) S^3_{rj}$$

$$\frac{dS^4_{rj}}{dt} = \theta S^3_{rj} + \sigma S^2_{rj} - (\pi^4_{rj} + \omega + \psi) S^4_{rj}$$

$$\frac{dH^1_{rj}}{dt} = \pi^1_{rj} S^1_{rj} + \pi^2_{rj} S^2_{rj} + \pi^3_{rj} S^3_{rj} + \pi^4_{rj} S^4_{rj} - (\nu^1 + \tau^T + \psi) H^1_{rj}$$

Force of infection

Individuals can form three different types of partnerships: (i) main partnerships (can be formed within or between all risk groups), (ii) non-main regular partnerships (can be formed within or between medium and high risk groups), and (iii) casual partnerships (can be formed only within the high risk group). In all of the following notation, j denotes a person's risk group, p denotes the risk group of the partner, and i denotes the type of partnership. The **force of infection,** π^x_{rj} (i.e. the probability that a susceptible acquires infection from their sexual partnerships, per unit time), is frequency-dependent and is defined separately for each subgroup, and for susceptibles not on PrEP ($x = 1$ for females/uncircumcised males, and $x = 3$ for circumcised males) and susceptibles on PrEP ($x = 2$ for females/uncircumcised males and $x = 4$ for circumcised males).

$$\pi^x_{rj} = \sum_{r'} \sum_{p} \sum_{i} \left[d_{rji} \rho_{rjpi} \sum_{s} \sum_{h} \left(\frac{I^{s,h}_{r'p}}{N_{r'p}} \varphi^x_{rjr'pish} \right) \right]$$

where

$\varphi^x_{rjr'pish}$ is the probability of transmission for a susceptible in subgroup (r, j) having a sexual partnership of type i with an individual from subgroup (r', p) who is in disease stage s (i.e. probability of infection per partnership per unit time), and depends on PrEP use/circumcision status (x) of the susceptible as well as ART/HTS status (h) of the infected individual

d_{rji} is the rate at which individuals in subgroup (r, j) form partnerships of type i with all other groups, per unit time

ρ_{rjpi} is the probability that individuals in subgroup (r, j) form sexual partnerships of type i with individuals in subgroup $(r', p) \rightarrow$ mixing parameter (controls the rate of partnership formation between specific subgroups)

$\frac{I^{s,h}_{r'p}}{N_{r'p}}$ is the proportion of individuals in risk group (r', p) that are infected and in disease stage s with ART/HTS status h

Then, $d_{rji}\rho_{rjpi}$ represents the total number of partnerships of type i that an individual from (r, j) forms with those from (r', p), per unit time. A correction for the resulting imbalance in partnerships is described below.

The **mixing parameters** ρ_{rjpi} represent the extent to which individuals within any behavioral subgroup form sexual partnerships of type i with individuals from different subgroups. Sexual mixing is assumed to be proportionate but with a tendency for within-class mixing,

$$\rho_{rjpi} = (1 - \mathcal{A}_i)\frac{d_{r'pi}n_{r'p}}{\sum_{\forall l} d_{r'li}n_{r'l}} + \mathcal{A}_i\zeta_{jp}$$

where

$\mathcal{A}_i$ is the degree of assortativity for sexual partnerships of type i

- $\mathcal{A}_i$ is assumed to be closer to 1 (i.e. assortative mixing) for main partnerships, and closer to 0 (i.e. random mixing) for non-main regular partnerships. For casual partnerships, mixing is always assortative ($\mathcal{A}_{casual} = 1$) since these can only be formed with individuals from subgroup $(r', 3)$.

ζ_{jp} is 1 if $j = p$ and 0 otherwise

n_{rj} is the total number of individuals in each subgroup

Hence, for main and non-main regular partnerships, a proportion $\mathcal{A}_i$ of partnerships are formed only with individuals from the same risk group. All others are formed with each risk group, proportional to the number of such partnerships offered by those risk groups.

The ρ_{rjpi} are defined for males and females from each sexual risk group, and for each type of partnership, e.g. $\rho_{F32_{reg}}$ denotes the level of mixing for non-main regular partnerships between females from the high risk group with males from the medium risk group. In order

to balance the number of partnerships between behavioral subgroups, the rates of partner acquisition (d_{rji}) are adjusted such that they will depend also on the behavioral subgroup of the partner (denoted by p) [5]. The degree of initial imbalance can be obtained as

$$B_{jpi} = \frac{d_{r'pi}\rho_{r'pji}n_{r'p}}{d_{rji}\rho_{rjpi}n_{rj}}$$

and equals 1 if the number of partnerships is balanced. B_{jpi} then represents the discrepancy in number of partnerships (of type i), which can be corrected by altering the partner acquisition rates of one or both sexes such that

$$d_{r'pji} = d_{r'pi}B_{jpi}^{x_i-1}$$

and

$$d_{rjpi} = d_{rji}B_{jpi}^{x_i}$$

where x_i is a constant controlling the extent to which each gender compromises with the wishes of the other gender (calibrated for each type of partnership). For example if both sexes were to compromise equally, $x_i = 0.5$. In this way, the following constraint on sexual mixing is met

$$d_{rjpi}\rho_{rjpi}N_{rj} = d_{r'pji}\rho_{rpji}N_{r'p}$$

The **transmission parameter** $\varphi^{x}_{rjr'pish}$ denotes the probability of transmission per partnership per unit time and depends on the susceptible individual's risk group (j) and PrEP/circumcision status (x), and the partner's risk group (p), disease stage (s) and HTS/ART use (h). It further depends on the type of partnership by the frequency of coital acts (η_{jpi}), probability of condom use per coital act (f_i), and efficacy of condom use (e_c). Condom use was modeled among the entire population, regardless of HIV status. The probability of transmission per coital act $\beta_r^{s,h,x}$ is defined in terms of the efficacy of ART (e_A), PrEP efficacy (e_P), level of PrEP adherence (P_r), efficacy of circumcision (e_{circ}), and the infected partner's disease stage (by a multiplication factor α). Hence, assuming a baseline $\beta_r^{1,1,1}$ for individuals in the chronic stage of infection that are not on ART/HTS and when the susceptible is not on PrEP, we obtain the following probabilities of transmission (see Table S1).

$$\beta_r^{2,1,1} = \alpha_1 \beta_r^{1,1,1}$$
$$\beta_r^{3,1,1} = \alpha_2 \beta_r^{1,1,1}$$
$$\beta_r^{4,1,1} = 0$$
$$\beta_r^{1,3,1} = \beta_r^{1,1,1}(1 - e_A)$$
$$\beta_r^{2,3,1} = \beta_r^{1,1,1}(1 - e_A)$$
$$\beta_r^{3,3,1} = \beta_r^{1,1,1}(1 - e_A)$$
$$\beta_r^{4,3,1} = \beta_r^{1,1,1}(1 - e_A)$$

$$\beta_r^{1,1,2} = \beta_r^{1,1,1}(1 - e_P P_r)$$
$$\beta_r^{2,1,2} = \beta_r^{2,1,1}(1 - e_P P_r)$$
$$\beta_r^{3,1,2} = \beta_r^{3,1,1}(1 - e_P P_r)$$
$$\beta_r^{4,1,2} = 0$$
$$\beta_M^{1,1,3} = \beta_M^{1,1,1}(1 - e_{circ})$$
$$\beta_M^{2,1,3} = \beta_M^{2,1,1}(1 - e_{circ})$$
$$\beta_M^{3,1,3} = \beta_M^{3,1,1}(1 - e_{circ})$$
$$\beta_M^{4,1,3} = 0$$

$$\beta_r^{1,3,2} = \beta_r^{1,3,1}(1 - e_P P_r)$$
$$\beta_r^{2,3,2} = \beta_r^{2,3,1}(1 - e_P P_r)$$
$$\beta_r^{3,3,2} = \beta_r^{3,3,1}(1 - e_P P_r)$$
$$\beta_r^{4,3,2} = \beta_r^{4,3,1}(1 - e_P P_r)$$
$$\beta_M^{1,3,3} = \beta_M^{1,3,1}(1 - e_{circ})$$
$$\beta_M^{2,3,3} = \beta_M^{2,3,1}(1 - e_{circ})$$
$$\beta_M^{3,3,3} = \beta_M^{3,3,1}(1 - e_{circ})$$
$$\beta_M^{4,3,3} = \beta_M^{4,3,1}(1 - e_{circ})$$

$$\beta_M^{1,3,4} = \beta_M^{1,3,3}(1 - e_P P_M)$$
$$\beta_M^{2,3,4} = \beta_M^{2,3,3}(1 - e_P P_M)$$
$$\beta_M^{3,3,4} = \beta_M^{3,3,3}(1 - e_P P_M)$$
$$\beta_M^{4,3,4} = \beta_M^{4,3,3}(1 - e_P P_M)$$
$$\beta_M^{1,1,4} = \beta_M^{1,1,3}(1 - e_P P_M)$$
$$\beta_M^{2,1,4} = \beta_M^{2,1,3}(1 - e_P P_M)$$
$$\beta_M^{3,1,4} = \beta_M^{3,1,3}(1 - e_P P_M)$$
$$\beta_M^{4,1,4} = 0$$

Then, the transmission parameters $\varphi^x_{rjr'pish}$ are as follows

- To women/uncircumcised men not on PrEP:

$$\varphi^1_{rjr'pish} = 1 - \left[(1 - \beta_r^{s,h,1} e_c)^{\chi_h} (1 - \beta_r^{s,h,1})^{\bar{\chi}_h} \right]$$

- To women/uncircumcised men on PrEP:

$$\varphi^2_{rjr'pish} = 1 - \left[(1 - \beta_r^{s,h,2} e_c)^{\chi_h} (1 - \beta_r^{s,h,2})^{\bar{\chi}_h} \right]$$

- To circumcised men not on PrEP:

$$\varphi^3_{MjFpish} = 1 - \left[(1 - \beta_M^{s,h,3} e_c)^{\chi_h} (1 - \beta_M^{s,h,3})^{\bar{\chi}_h} \right]$$

- To circumcised men on PrEP:

$$\varphi^4_{MjFpish} = 1 - \left[(1 - \beta_M^{s,h,4} e_c)^{\chi_h} (1 - \beta_M^{s,h,4})^{\bar{\chi}_h} \right]$$

where

$\beta_r^{s,h,x}$ is the transmission probability corresponding to the partner's disease stage, ART use and susceptible's PrEP/circumcision status (Table S1)

χ_h ($\bar{\chi}_h$) denotes the proportion of coital acts (not) protected by condom use ($h = 1$ if no HTS/ART, $h = 2$ if HTS, $h = 3$ if ART), where, for $h = 1$ and $h = 3$,

$$\chi_h = f_i \eta_{jpi}$$

and

$$\bar{\chi}_h = (1 - f_i)\eta_{jpi}$$

In case the infected partner is aware of their status (i.e. assumed to reduce risk behaviour due to HIV counseling, $h = 2$),

$$\chi_2 = f_i \xi \eta_{jpi} \epsilon$$

and

$$\bar{\chi}_2 = (1 - f_i \xi) \eta_{jpi} \epsilon$$

where ξ denotes the increase in condom use due to HTS, and ϵ denotes a decrease in the frequency of coital acts.

Intervention uptake rates

The rate of diagnosis τ^T is modelled such that the proportion of infected individuals that are aware of their status increases linearly over time until reaching a specified level P_{HTS} in 2014. This is achieved as follows [6]:

- if $t < t_{start}$

$$\tau^T(t) = 0$$

- if $t_{start} < t \leq t_{start} + \frac{1}{r_T}$

$$\tau^T(t) = \left[(t - t_{start}) r_T P_{HTS}\right] - \left[\frac{I^2(t) + I^3(t)}{I(t)}\right]$$

where t_{start} is the time at which HIV testing is assumed to start (i.e. 1987), $1/r_T$ is the time it takes to reach the desired baseline coverage level P_{HTS} in 2014, and $\left[\frac{I^2(t)+I^3(t)}{I(t)}\right]$ is the proportion aware of their HIV+ status at time t. In our baseline model (i.e. the model run for an additional 15 years without intervention scale-up), P_{HTS} is slightly increased to 65% to avoid a zero rate of treatment uptake. In the model with intervention scale-up, the rate of diagnosis is increased from 2014 onward such that the proportion aware of their HIV+ status in 2029 reaches a specified level P_{HTS2} as

- if $t > t_{start} + \frac{1}{r_T}$

$$\tau^T(t) = \tau^T(t-1) + \left[\left(t - (t_{start} + \frac{1}{r_T})\right) r_{T2} (P_{HTS_2} - P_{HTS})\right] - \left[\frac{I^2(t) + I^3(t)}{I(t)} - pHTS\right]$$

where $1/r_{T2}$ is the duration of scale-up.

A proportion of individuals, P_{ART}, aware of their HIV-positive status initiate ART on average one year after diagnosis, resulting in a monthly ART uptake rate of

$$\tau_A^s(t) = \Big(1 + (-log(1 - P_{ART}(t)))\Big)^{\frac{1}{12}} - 1$$

where P_{ART} is assumed to increase over time according to a Hill function until reaching a specified baseline value.

$$P_{ART} = \begin{cases} 0 & \text{if } t < t_1 \\ P_{max}^{art} \frac{(t-t_1)^2}{(t-t_1)^2+10^2} & \text{if } t_1 \le t \le t_2 \\ P_{max}^{art} + (P_{max.up}^{art} - P_{max}^{art}) \frac{(t-t_2)^6}{(t-t_2)^6+95^6} & \text{if } t > t_2 \end{cases}$$

where P_{max}^{art} is the desired probability of ART initiation at t_2 (i.e. 2014), and $P_{max.up}^{art}$ is the desired probability of ART initiation after scale-up (i.e. 2029). For infected individuals in the acute and chronic stages of infection $t_1 = 2011$, while for those in the (pre-)AIDS stage $t_1 = 2004$.

Before 2014, the rate of HTS uptake in susceptible individuals is assumed to be equal to the rate of diagnosis, hence the rate of VMMC uptake before 2014 and in our baseline scenario is

$$\sigma(t) = \left[\Big(1 + (-log(1 - P_{CIRC}(t)))\Big)^{\frac{1}{12}} - 1\right] HTS_m \tau^T(t)_{\{t \le 2014\}}$$

where HTS_m represents reduced HTS uptake in males and P_{CIRC} is the proportion of individuals accepting VMMC on average one year after being tested. As such we assume there was no VMMC before the introduction of HIV testing, although a constant proportion p_c of males enter the population already circumcised. The probability P_{CIRC} of accepting VMMC increases over time according to a Hill function until reaching a specified value.

$$P_{CIRC} = \begin{cases} 0 & \text{if } t < t_1 \\ P_{max}^{circ} \frac{(t-t_1)^4}{(t-t_1)^4+100^4} & \text{if } t_1 \le t \le t_2 \\ P_{max}^{circ} + (P_{max.up}^{circ} - P_{max}^{circ}) \frac{(t-t_2)^6}{(t-t_2)^6+85^6} & \text{if } t > t_2 \end{cases}$$

where P_{max}^{circ} is the desired probability of accepting VMMC at t_2 (i.e. 2014), and $P_{max.up}^{circ}$ is the desired probability of accepting VMMC after scale-up (i.e. 2029). In the models with intervention scale-up, HTS uptake in susceptible individuals is given by

$$\tau^{ST}(t) = P_{HTS}^S \frac{(t - t_1)^5}{(t - t_1)^5 + 100^5}$$

where P^S_{HTS} is the desired proportion of susceptible individuals accepting HTS after intervention scale-up, and $t_1 = 2014$. The rate of VMMC uptake after 2014 then becomes

$$\sigma(t) = \left[\left(1 + (-log(1 - P_{CIRC}(t)))\right)^{\frac{1}{12}} - 1\right] HTS_m \tau^{ST}(t).$$

PrEP uptake is only included in the model with intervention scale-up and is modelled similarly, with P_{PrEP} being the proportion of susceptible individuals initiating PrEP on average one year after testing, which increases over time until reaching a specified level P^{prep}_{max},

$$P_{PrEP} = \begin{cases} 0 & \text{if } t < t_1 \\ P^{prep}_{max} \frac{(t-t_1)^4}{(t-t_1)^5 + 85^5} & \text{if } t \geq t_1 \end{cases}$$

where $t_1 = 2015$. The rate of PrEP uptake then becomes

$$\theta_M(t) = \left[\left(1 + (-log(1 - P_{PrEP}(t)))\right)^{\frac{1}{12}} - 1\right] HTS_m \tau^{ST}(t)$$

for males, and

$$\theta_F(t) = \left[\left(1 + (-log(1 - P_{PrEP}(t)))\right)^{\frac{1}{12}} - 1\right] \tau^{ST}(t)$$

for females.

Condom use is assumed to be different for each type of partnership and increases over time according to a Hill function

$$f_i(t) = \begin{cases} f_{i_{max}} \frac{(t-0)^4}{(t-0)^4 + 200^4} & \text{if } t < t_1 \\ f_{i_{max}} + (f_{i_{max.up}} - f_{i_{max}}) \frac{(t-t_1)^6}{(t-t_1)^6 + 85^6} & \text{if } t \geq t_1 \end{cases}$$

where $f_{i_{max}}$ is the maximum (baseline) level of condom use. After 2014, $f_{i_{max}}$ is increased to $f_{i_{max.up}}$ representing intervention scale-up.

Model calibration

The model was run for 45 years using a time step of one month for each of the 10 000 LHS-generated parameter input sets θ obtained from the uncertainty ranges in Table S2. Overall goodness of fit (GOF) was measured as the sum of the squared deviations from the target prevalence in each risk group, p_{rj}, and the total prevalence p,

$$GOF = (\hat{p} - p)^2 + \sum (\hat{p_{rj}} - p_{rj})^2$$

We used an active learning approach to reduce the uncertainty ranges of the model parameters in an iterative way. After each iteration, the subspace of parameters leading to the top 1% of model outputs (in terms of GOF), $\Theta_{1\%}$ was investigated using several methods and where possible the uncertainty ranges were reduced.

The activity region finder (ARF) proposed by Amaratunga and Cabrera was first used to investigate the subspace $\Theta_{1\%}$.[7] ARF is a recursive partitioning classification tree method where each of the 10 000 LHS-generated parameter sets are considered as input, and their corresponding binary indicators that equal 1 if $\theta \in \Theta_{1\%}$ and 0 otherwise are considered as output. ARF then shows which input parameter sets occur more frequently in the subspace $\Theta_{1\%}$ and thus provide a better fit to the observed prevalence data. The maximal information coefficient (MIC) was then used to quantify the association structure between the parameters in the subspace $\Theta_{1\%}$.[8] The MIC ranges between 0 and 1, with higher values indicating a stronger relationship between the parameters. In this way, insight was obtained on which parameters are highly correlated and thus would be more difficult to estimate. Additionally, influence of the data on the estimated parameters was identified by univariately exploring the subspace $\Theta_{1\%}$. For each parameter a graphical comparison of the (prior) density of the uniform distribution f_U (i.e. the range as in Table S2) with the (posterior) density $f_{\Theta_{1\%}}$, corresponding to the values within the subspace $\Theta_{1\%}$, was made.[9] A peaked unimodal density $f_{\Theta_{1\%}}$ indicates that the parameter is (highly) informed by the data. In case a parameter's updated uncertainty range would be small and the parameter appeared to be not highly informed by the data, this parameter was fixed to its value in the current 'best' solution. This active learning process was repeated until an optimal value for the parameters was obtained or until the parameter ranges were stable in case they could not be reduced to a single value.

For most parameters, stability in uncertainty ranges was achieved after 24 iterations, and the best GOF (lowest discrepancy between observed and simulated prevalence) was obtained. Model parameters corresponding to the 'best fit' model are shown in Table S2. In the final iteration, the highest MIC was 0.56, measuring the association between η_{33main} (frequency of main partnership coital acts among individuals from the high risk group) and x_{casual} (constant controlling the extent to which each gender compromises with the wishes of the other gender, for casual partnerships). MIC was also high between β_m and P_{HTS} (0.50) and between f_{casual} and η_{33main} (0.46). All other MIC were smaller than 0.40, indicating no substantial association between the remaining parameters in the

subspace $\Theta_{1\%}$. The unimodal peaks in Figure S2 show that some of these parameters with high MIC also appear to be quite informed by the data. In addition, these parameters also often took part in the ARF process, further indicating the difficulty in reducing their ranges. Figure S3 shows the evolution of HIV prevalence (a) and incidence (b) under the 'best fit' model without increasing intervention uptake after 2014.

Assessing the impact of intervention scale-up

As baseline, we run the calibrated model for an additional 15 years (2014-2029) without intervention scale-up (i.e. assuming uptake remains constant from 2014 onward). To assess the impact of different combinations of increasing intervention uptake, we use LHS to sample 500 input parameter sets θ_I from the ranges of the intervention parameters (Table S3). We first investigate the impact of intervention scale-up by running the 'best fit' model for an additional 15 years (2014-2029). Different combinations of intervention scale-up were compared in terms of their impact on the relative reduction in HIV incidence and prevalence, AIDS-related mortality, and the total number of new infections, by comparing the model with intervention scale-up to the baseline model without increased intervention uptake. To account for the remaining uncertainty in the baseline model parameters, parameter ranges of simulated epidemics belonging to the top 1% of model outputs, $\Theta_{1\%}$, were also used to investigate the impact of combined interventions.

Intervention impact was evaluated on the following model output (as % reduction), per subgroup (r, j) and overall.

Prevalence: $P_{rj}(t) = \frac{\sum_{h=1}^{3}(H_{rj}^h(t)+Y_{rj}^h(t)+Z_{rj}^h(t)+A_{rj}^h(t))}{N_{rj}}$

New infections: $NI_{rj}(t) = \sum_x \pi_{rj}^x(t) S_{rj}^x(t)$

Incidence (per 100 PY): $IR(t) = \frac{\sum_{x,r,j} \pi_{rj}^x S_{rj}^x}{\sum_{x,r,j} S_{rj}^x} \times 100 \times 12$

AIDS-related mortality (per 100 PY): $\Delta_{rj}(t) = \frac{\sum_{h=1}^{3}(\delta^h A_{rj}^h(t))}{N_{rj}(t)} \times 100 \times 12$

B Supplementary Tables

Table S1. Probability of transmission (β) per coital act, where baseline corresponds to the chronic low viral load (LV) stage (r = gender indicator).

Disease stage	ART	ART+PrEP	ART+CIRC	ART+PrEP+CIRC	PrEP	CIRC	CIRC+PrEP
Baseline: $\beta_r^{1,1,1}$	$\beta_r^{1,3,1}$	$\beta_r^{1,3,2}$	$\beta_M^{1,3,3}$	$\beta_M^{1,3,4}$	$\beta_r^{1,1,2}$	$\beta_M^{1,1,3}$	$\beta_M^{1,1,4}$
Initial HV: $\beta_r^{2,1,1}$	$\beta_r^{2,3,1}$	$\beta_r^{2,3,2}$	$\beta_M^{2,3,3}$	$\beta_M^{2,3,4}$	$\beta_r^{2,1,2}$	$\beta_M^{2,1,3}$	$\beta_M^{2,1,4}$
pre-AIDS LV: $\beta_r^{3,1,1}$	$\beta_r^{3,3,1}$	$\beta_r^{3,3,2}$	$\beta_M^{3,3,3}$	$\beta_M^{3,3,4}$	$\beta_r^{3,1,2}$	$\beta_M^{3,1,3}$	$\beta_M^{3,1,4}$
AIDS HV: $\beta_r^{4,1,1}$	$\beta_r^{4,3,1}$	$\beta_r^{4,3,2}$	$\beta_M^{4,3,3}$	$\beta_M^{4,3,4}$	$\beta_r^{4,1,2}$	$\beta_M^{4,1,3}$	$\beta_M^{4,1,4}$

Table S2. Uncertainty ranges and fixed values for the baseline model parameters and value in the 'best fit' solution.

Parameter	Range / Value Male	Range / Value Female	Reference	Best fit Male	Best fit Female
**Demographic**					
Initial population size (N)	2000				
Population size per class					
low risk	0.33*N	0.43*N	HIVCOMB 2014 census [10]		
medium risk	0.07*N	0.02*N	HIVCOMB 2014 census [10]		
high risk	0.10*N	0.05*N	HIVCOMB 2014 census [10]		
Mortality/recruitment rate (ψ)	0.0019		Based on 45 years of sexual activity		
Population growth rate (μ)	0.0021		Based on yearly growth rate of 2.5%		
**Epidemiologic**					
Initial HIV prevalence	1%				
Untreated disease progression					
Average duration of initial HV stage ($1/\nu^1$)	3.25 months		[11]		
Average duration of LV stage ($1/\Omega^1$)	100 months				
Average duration of pre-AIDS stage ($1/\Psi^1$)	14 months				
Average duration of AIDS stage ($1/\delta^1$)	16 months				
Treated disease progression					
Average duration of initial HV stage ($1/\nu^2$)	98 months		[11]		
Average duration of LV stage ($1/\Omega^2$)	648 months				
Average duration of pre-AIDS stage ($1/\Psi^2$)	30 months				
Average duration of AIDS stage ($1/\delta^2$)	33 months				
**Transmission**					
Transmission probability per sex act (β_r)	$0.001 - 0.003$		[12]	0.001	0.002
Multiplication factor (treated = untreated)					
initial HV (α_1)	26		[13]		
pre-aids HV (α_2)	7		[13]		
AIDS stage (α_3)	1				
**Intervention**					
Efficacy of ART (e_a)	0.90		Reduction in infectiousness [14]		
Efficacy of PrEP (e_p)	0.67		67% reduction in susc. [15]		
PrEP adherence (P_r)	0.8		Assumed		
Efficacy of condom use (e_c)	0.9		Relative infectiousness of 0.1 [14]		
Efficacy of circumcision (e_{circ})	0.60		Reduction in susceptibility [16]		
Rate of PrEP uptake (θ)	0		No PrEP in baseline model		
Rate of PrEP discontinuation (ω)	0		No PrEP in baseline model		
Maximum probability of accepting ART (P^{art}_{max})					
Acute stage	0.05 - 0.3		Assumed	0.157	
Chronic stage	0.05 - 0.3		Assumed	0.192	
Pre-AIDS stage	0.1 - 0.3		Assumed	0.185	
AIDS stage	0.2 - 0.6		Twice as fast as in pre-AIDS stage	0.371	
Rate of ART dropout (γ)	0.0186		Annual dropout of 20% [17]		
Proportion accepting VMMC (P^{circ}_{max})	0.1 - 0.5	NA	Assumed annual proportion of 10-50%	0.313	NA
Proportion already circumcised (p_c)	0.1 - 0.2	NA	[18]	0.318	NA
Proportion aware of HIV+ status in 2014 (P_{HTS})	0.5 - 0.7		Assumed	0.634	
Relative HTS uptake in males	0.5 - 0.9	NA	[19]	0.700	NA
Relative ART uptake in males	0.5 - 0.95	NA	[19]	0.759	NA
Relative number of sex acts due to HTS (ϵ)	0.9		Assume 10% reduction		
Increase in condom use due to HTS (ξ)	1.10		Assume 10% increase		
Relative number of sex acts due to untreated AIDS (ϵ_{AIDS})	0.1		Assumed		
**Behavioral**					
Probability of condom use per sex act ($f_{i_{max}}$)					
Main partnerships	0.01 - 0.1		Assumed	0.070	
Non-main regular partnerships	0.1 - 0.3		Assumed	0.261	
Casual partnerships	0.3 - 0.6		HIVCOMB 2014 census [10]	0.428	
Number of casual sex acts per month ($\eta_{rj_{casual}}$)					
high-high risk	1-5		Assumed	3.586	
Number of non-main regular sex acts per month ($\eta_{rj_{regular}}$)					
medium-medium risk	1-5		Assumed	4.338	
medium-high risk	1-5		Assumed	5.535	
high-medium risk	1-5		Assumed	8.429	
high-high risk	1-5		Assumed	2.587	
Number of main sex acts per month ($\eta_{rj_{main}}$)					

low-low risk	1-30	Assumed	19.843	
low-medium risk	1-30	Assumed	16.364	
low-high risk	1-30	Assumed	8.925	
medium-low risk	1-20	Assumed	6.471	
medium-medium risk	1-20	Assumed	4.834	
medium-high risk	1-20	Assumed	5.835	
high-low risk	1-15	Assumed	5.676	
high-medium risk	1-15	Assumed	4.808	
high-high risk	1-15	Assumed	5.469	
Rate of partner acquisition for main partnerships ($d_{rj_{main}}$)				
low risk	0.0083 - 0.4167	Assuming 5 new main partners per year to one every 10 years	0.114	0.347
medium risk	0.0083 - 0.4167	Assuming 5 new main partners per year to one every 10 years	0.339	0.266
high risk	0.0083 - 0.4167	Assuming 5 new main partners per year to one every 10 years	0.187	0.225
Rate of partner change for regular partnerships ($d_{rj_{regular}}$)				
medium risk	0.4167 - 0.8333	Assuming 5-10 new partners every year	0.708	0.604
high risk	0.4167 - 0.8333	Assuming 5-10 new partners every year	0.547	0.571
Rate of partner change for casual partnerships ($d_{rj_{casual}}$)				
high risk	0.6667 - 1	Assuming 8-12 new partners every year	0.944	0.766
Degree of assortativity ($\mathcal{A}_i$)				
Main partnerships	$0.5 - 0.9$	Assumed tendency towards assortative mixing	0.573	
Non-main regular partnerships	$0.1 - 0.5$	Assumed tendency towards random mixing	0.216	
Casual partnerships	1	Motivated in text		
Constant to adjust partnership imabalance (x_i)				
Main partnerships	0.2 - 0.8	Assumed	0.301	
Non-main regular partnerships	0.2 - 0.8	Assumed	0.394	
Casual partnerships	0.2 - 0.8	Assumed	0.201	

Table S3. Uptake ranges for the intervention parameters and their values in the highest impact scenario.

Intervention	Range / Value (Male)	Range / Value (Female)	Reference	Highest impact scenario	Baseline model
Efficacy of PrEP (e_p)	0.67		67% reduction in susc. [15]		
PrEP adherence (P_r)	0.6		Assumed		
Probability of initiating PrEP (P^{prep}_{max})	0.2 - 0.8		Assumed	0.3040	NA
Proportion of susceptibles accepting HTS (P^{S}_{HTS})	0.2 - 0.8		Assumed	0.4615	NA
Rate of PrEP discontinuation (ω)	0.0186		Annual dropout of 20% (assumed)		
Probability of initiating ART ($P^{art}_{max.up}$)					
Acute stage	0.2 - 0.8		Assumed	0.3315	0.157
Chronic stage	0.2 - 0.8		Assumed	0.7251	0.192
Pre-AIDS stage	0.2 - 0.8		Assumed	0.3985	0.185
AIDS stage	0.4 - 0.8		Assumed	0.5197	0.371
Rate of ART dropout (γ)	0.0186		Annual dropout of 20% [17]		
Proportion accepting VMMC ($P^{circ}_{max.up}$)	NA	0.3 - 0.8	Assumed	0.6305	0.313
Proportion aware of HIV+ status in 2029 (P_{HTS_2})	0.6 - 0.9		Assumed	0.8944	NA
Probability of condom use per sex act ($f_{i_{max.up}}$)					
Main partnerships	0.1 - 0.6		Assumed	0.5806	0.070
Non-main regular partnerships	0.3 - 0.6		Assumed	0.5818	0.261
Casual partnerships	0.5 - 0.6		Assumed	0.585	0.428

Table S4. Sensitivity analyses for the impact of combined intervention.

Scenario	Relative reduction compared to baseline over 15 years (2014 - 2029)			
	Incidence	Prevalence	AIDS-related mortality	Number of new infections
PrEP adherence of 90%	0.8680	0.3815	0.5378	0.3987
PrEP adherence of 30%	0.8657	0.3805	0.5374	0.4119
PrEP dropout rate of 5%	0.8676	0.3812	0.5377	0.4125
No PrEP use	0.8651	0.3802	0.5373	0.3976
No increase in condom use	0.5619	0.1559	0.4111	0.2063
ART dropout rate of 5% from 2014 onward	0.9116	0.3915	0.6368	0.4751
ART dropout rate of 70% from 2014 onward	0.7778	0.3637	0.3407	0.2999
ART dropout rate of 95% from 2014 onward	0.7383	0.3576	0.2521	0.2546
Equal ART and HTS/SBCC uptake in males and females	0.8811	0.3980	0.5647	0.4421
Probability of 30% of ART initiation in chronic stage	0.8154	0.3783	0.4154	0.3805

C Supplementary Figures

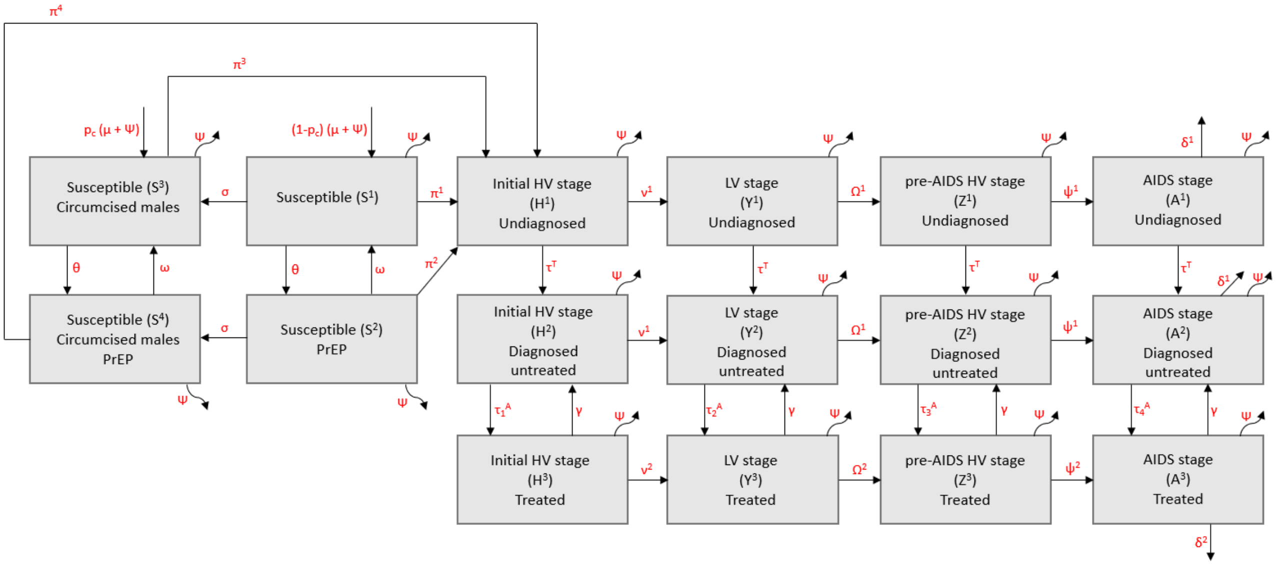

Figure S1: Model structure for males in risk group j (which is the same for females, without circumcision compartments); HV = high viral load, LV = low viral load. Notation: π = force of infection; p_c = proportion of males entering the population already circumcised; ω = PrEP discontinuation rate; θ = PrEP uptake rate; ψ = non-AIDS related mortality rate; σ = rate of VMMC uptake; μ = 'birth' rate; ν = 1 / (duration of HV stage); τ = rate of ART initiation; γ = rate of ART dropout; Ω = 1 / (duration of LV stage); Ψ = 1 / (duration of pre-AIDS HV stage); δ = AIDS-related mortality rate

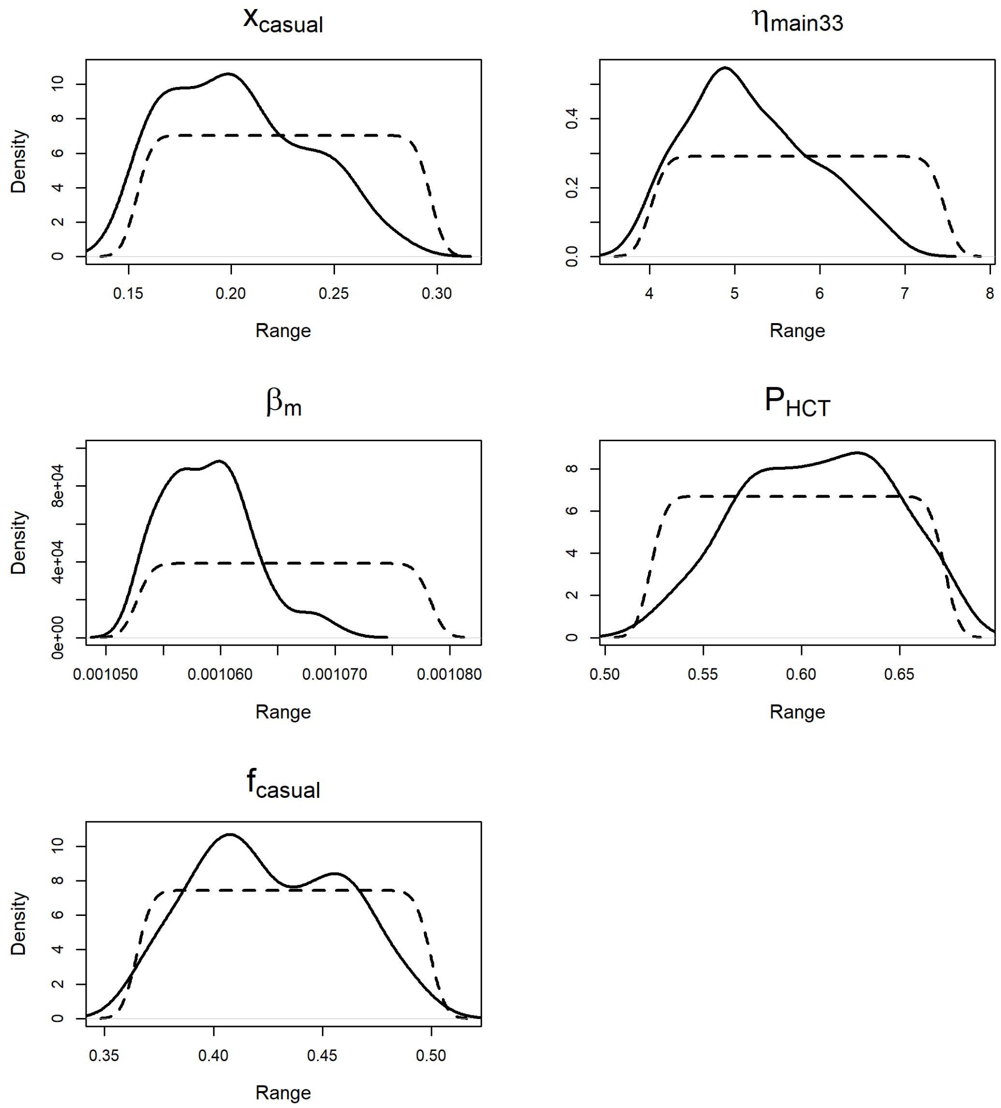

Figure S2: Parameters that are highly informed by the observed prevalence data. Dashed lines represent the density f_U of the uniform distribution on the uncertainty range of the input parameter. Solid lines represent the density $f_{\Theta 1\%}$ corresponding to the values within the subspace $\Theta_{1\%}$. A peaked unimodal density $f_{\Theta 1\%}$ indicates that the parameter was highly informed by the data. x_{casual} = constant to adjust partnership imbalance for casual partnerships; η_{main33} = number of main sex acts per month among individuals in the high-risk group; β_m = male-to-female transmission probability; P_{HTS} = proportion aware of HIV-positive status; f_{casual} = condom use in casual relationships.

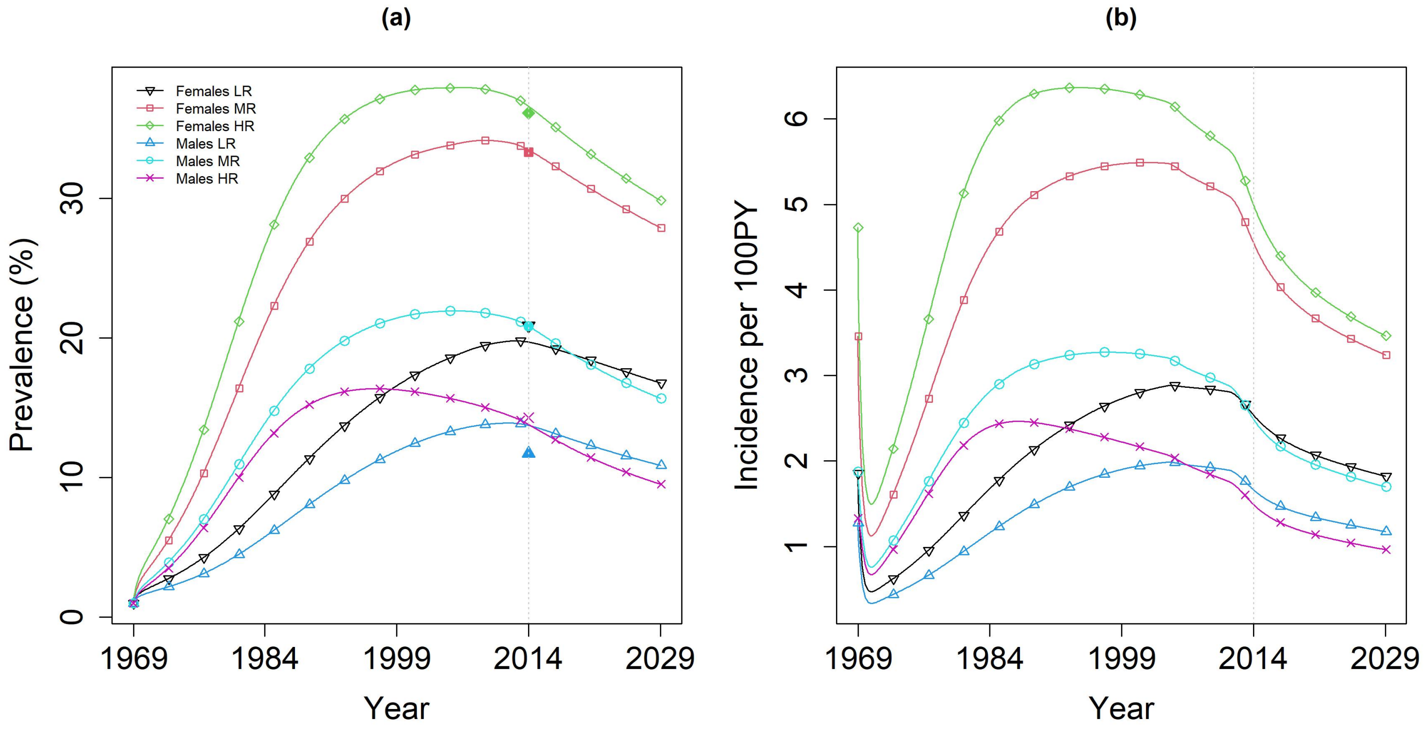

Figure S3: HIV prevalence (a) and incidence (b) in the different sexual risk groups obtained from the 'best fit' model, 1969-2029. Filled symbols on the dotted grey vertical line represent the observed prevalence in 2014 obtained from the HIVCOMB study [10], to which the model was calibrated. LR = low risk, MR = medium risk, HR = high risk.

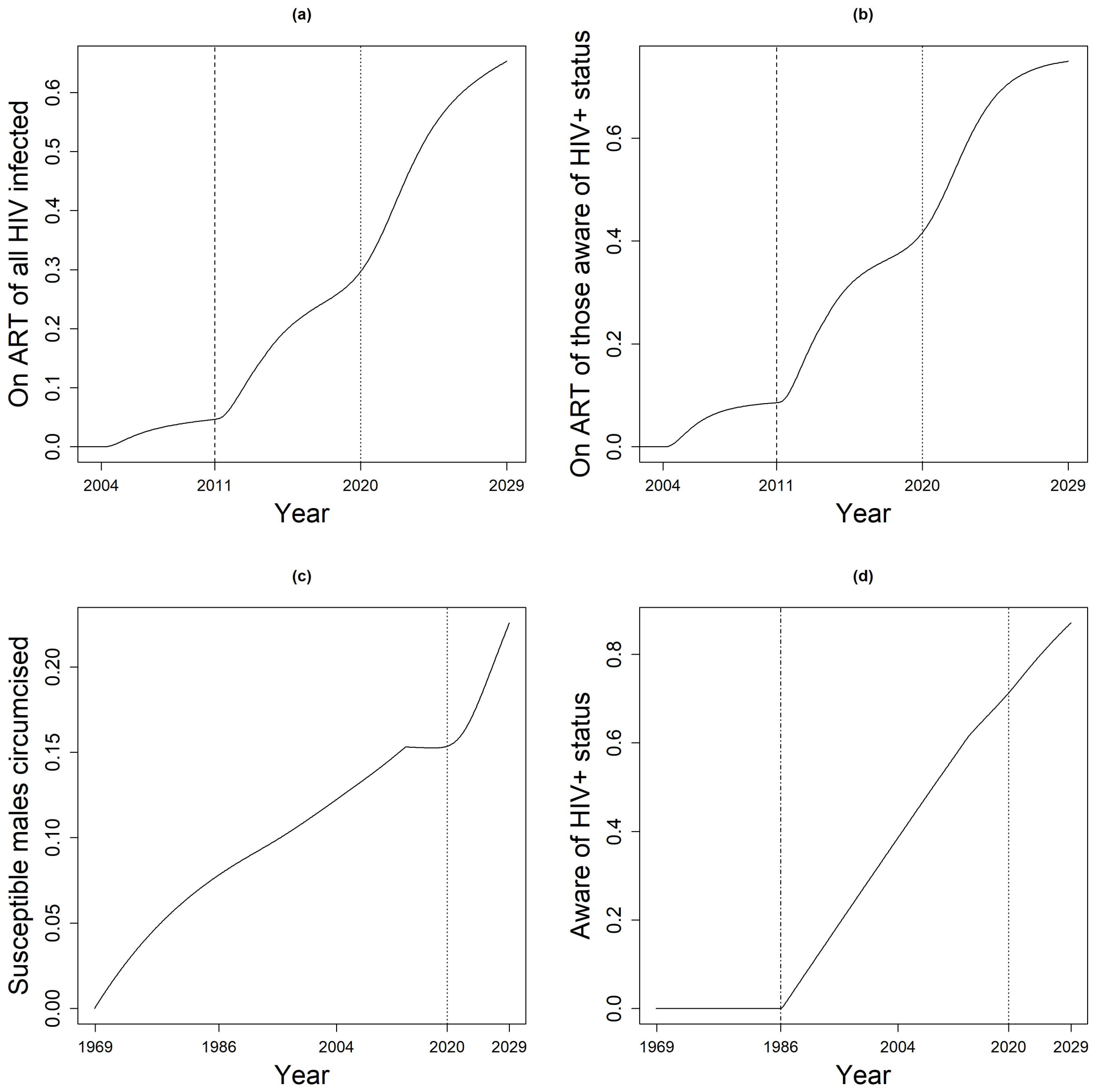

Figure S4: Intervention coverage over time. (a) HIV infected individuals on ART, (b) HIV infected individuals aware of their status on ART, (c) circumcised susceptible males, and (d) individuals aware of their HIV+ status. Dashed lines indicate the year 2011 when ART was implemented in the chronic stage, dotted lines indicate the year 2020 when intervention scale-up was started, dash-dotted lines indicate 1987 when HIV testing was introduced.

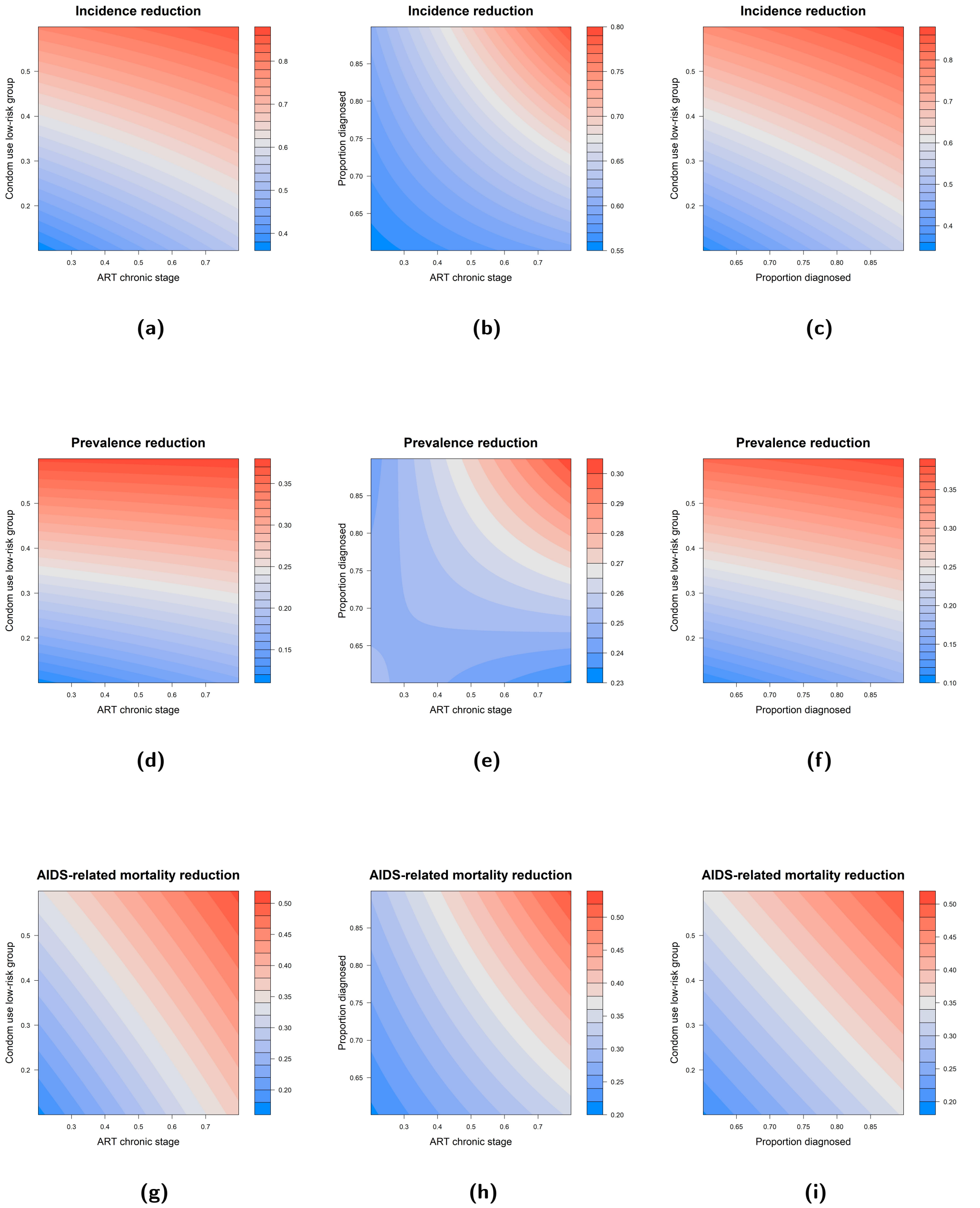

Figure S5: Simultaneous effect of combinations of the three most important interventions on the relative reduction in incidence (a, b, c), prevalence (d, e, f), and AIDS-related mortality (g, h, i).

References

[1] Uganda AIDS Commission. National HIV and AIDS strategic plan 2015/2016-2019/2020. 2015.

[2] T. S. Alexander. Human immunodeficiency virus diagnostic testing: 30 years of evolution. *Clinical and Vaccine Immunology*, 23:249–253, 2016.

[3] Ministry of Health Uganda. Uganda national antiretroviral treatment and care guidelines for adults, adolescents, and children. 2003.

[4] R. Mafigiri, J. K. B. Matovu, F. E. Makumbi, A. Ndyanabo, D. Nabukalu, M. Sakor, G. Kigozi, F. Nalugoda, and R. K. Wanyenze. HIV prevalence and uptake of HIV/AIDS services among youths (15-24 years) in fishing and neighboring communities of Kasensero, Rakai District, South Western Uganda. *BMC Public Health*, 17:251, 2017.

[5] G. P. Garnett and R. M. Anderson. Balancing sexual partnerships in an age and activity stratified model of HIV transmission in heterosexual populations. *IMA Journal of Mathematics Applied in Medicine & Biology*, 11:161–192, 1994.

[6] I. Cremin, R. Alsallaq, M. Dybul, P. Piot, G. Garnett, and T. B. Hallet. The new role of antiretrovirals in combination HIV prevention: a mathematical modelling analysis. *AIDS*, 27:447–458, 2013.

[7] D. Amaratunga and J. Cabrera. Mining data to find subsets of high activity. *Journal of Statistical Planning and Inference*, 122:23–41, 2004.

[8] D. N. Reshef, Y. A. Reshef, and H. K. Finucane et al. Detecting novel associations in large data sets. *Science*, 334:1518–1524, 2011.

[9] A. Y. Castro Sanchez, M. Aerts, Z. Shkedy, P. Vickerman, F. Faggiano, G. Salamina, and N. Hens. A mathematical model for HIV and hepatitis C co-infection and its assessment from a statistical perspective. *Epidemics*, 5:56–66, 2013.

[10] M. O. Kuteesa, H. A. Weiss, A. Abaasa, S. Nash, R. N. Nsubuga, R. Newton, J. Seeley, and A. Kamali. Feasibility of conducting hiv combination prevention interventions in fishing communities in uganda: a pilot cluster randomized trial. *PLoS ONE*, 14, 2019.

[11] G. Rozhnova, M. Anastasaki, and M. Kretzschmar. Modelling the dynamics of population viral load measures under HIV treatment as prevention. *Infectious Disease Modelling*, 3:160–170, 2018.

[12] M. C. Boily, R. F. Baggaley, L. Wang, B. Masse, R. G. White, R. Hayes, and M. Alary. Heterosexual risk of HIV-1 infection per sexual act: a systematic review and meta-analysis of observational studies. *Lancet Infectious Diseases*, 9:118–129, 2009.

[13] T. D. Hollingsworth, R. M. Anderson, and C. Fraser. HIV-1 transmission, by stage of infection. *Journal of Infectious Diseases*, 198:687–693, 2008.

[14] Jinghua Li, Liping Peng, Stuart Gilmour, Jing Gu, Yuhua Ruan, Huachun Zou, Chun Hao, Yuantao Hao, and Joseph Tak-fai Lau. A mathematical model of biomedical interventions for hiv prevention among men who have sex with men in china. *BMC infectious diseases*, 18(1):1–9, 2018.

[15] J. M. Baeten, D. Donnell, and P. Ndase et al. Antiretroviral prophylaxis for HIV prevention in heterosexual men and women. *The New England Journal of Medicine*, 367:399–410, 2012.

[16] R. H. Gray, G. Kigozi, and D. Serwadda et al. Male circumcision for HIV prevention in men in Rakai, Uganda: a randomized trial. *Lancet*, 369:657–666, 2007.

[17] Uganda AIDS Commission et al. Hiv and aids uganda country progress report. 2014; kampala, uganda: Uganda aids commission.

[18] Uganda Bureau of Statistics. The national population and housing census 2014 - main report. 2016.

[19] UPHIA. Uganda population based hiv impact assessment uphia 2016-2017, 2017.
